# Licoisoflavone B and glabridin from *Glycyrrhiza glabra* as potent nucleoprotein antagonists of Lassa virus: insights from molecular docking, dynamics simulation, PCA, and DFT studies

**DOI:** 10.1016/j.jgeb.2025.100544

**Published:** 2025-08-05

**Authors:** Sk. Faisal Ahmed, Md. Masudur Rahman Munna, Md. Hossain Ahmed, Md. Mostafizur Rahman, Minhajul Islam, Esha Mony Bristy

**Affiliations:** a Department of Biochemistry and Microbiology, North South University, Dhaka, Bangladesh; b Dawn of Bioinformatics Limited, Dhaka, Bangladesh; c Department of Biochemistry and Molecular Biology, Bangladesh Agricultural University, Mymensingh 2202, Bangladesh; d Department of Biotechnology and Genetic Engineering, Gopalganj Science and Technology University, Gopalganj 8100, Bangladesh; e Department of Biotechnology and Genetic Engineering, Jahangirnagar University, Dhaka 1342, Bangladesh; f BBA in Management, Bangladesh National University, Gazipur, Bangladesh

**Keywords:** Lassa fever, Viral replication, ADMET, Molecular dynamics simulation, Principal component analysis

## Abstract

Lassa virus causes a severe hemorrhagic disease referred to as Lassa fever. It exhibits a significant mortality rate among people in West and Central Africa. Currently, there is no vaccine available, and ribavirin is the sole treatment option with significant limitations. Given the lack of an effective medication, this study explores bioactive phytocompounds from the plant *Glycyrrhiza glabra* that may be safer and more effective than ribavirin in combating viruses’ nucleoprotein activity, which is essential for the replication of viruses and host immune evasion. Our virtual screening and multi-stage molecular docking analyses of 69 natural phytochemicals from this plant revealed the compounds licoisoflavone B and glabridin as potential therapeutics. These compounds exhibit strong binding affinities toward the target protein, with superior ADMET profiles. Both compounds also maintained structural stability throughout 100 ns molecular dynamics simulations, while principal component analysis further corroborated their conformational stability, highlighting potential efficiency. Furthermore, density functional theory analysis indicated favorable electronic properties, supporting the compounds’ potential as viable drug candidates. These findings suggested licoisoflavone B and glabridin as potential therapeutic candidates for Lassa fever. However, this study underscores the urgency of further experimental validation to advance these compounds toward novel anti-Lassa virus therapeutics.

## Abbreviations

**Abbreviation**: **Definition**
LASV: Lassa Virus
LF: Lassa Fever
NP: Nucleoprotein
PDB: Protein Data Bank
ADMET: Absorption, Distribution, Metbolism, Excretion, and Toxicity
MD: Molecular Dynamics
PCA: Principal Component Analysis
CID: Compound Identification Number
MM_PBSA: Molecular Mechanics/Poisson–Boltzmann Surface Area
HOMO: Highest Occupied Molecular Orbital
LUMO: Lowest Unoccupied Molecular Orbital
DFT: Density Functional Theory
CADD: Computer Aided Drug Design

## 1 Introduction

Lassa virus (LASV), an RNA virus from the Arenaviridae family, is endemic to West African nations and causes human hemorrhagic Lassa fever (LF).^1^, ^2^ LASV transmission is primarily through rodent-to-human propagation but also from person to person. Travel has led to the transfer of LF scenarios from endemic Western African regions to non-endemic areas, where the disease is not typically present.^3^ In 1969, the African nation of Nigeria recorded the first officially documented example of the Lassa virus, with yearly reports indicating a prevalence of 100,000–300,000 cases, leading to around 5,000 deaths. Lassa fever represents a significant concern, contributing 10 to 16 percent of hospitalizations throughout Liberia and Sierra Leone, largely influenced by geographical disparities in disease surveillance practices. Between the first week and the 52nd week of 2022, Nigeria experienced a significant increase in LASV cases, with at least 1,067 identified patients throughout 27 states and 112 local government areas (LGAs), resulting in 189 deaths. Between December 2022 and January 2023, the Nigeria Centre for Disease Control and Prevention (NCDC) documented 8,202 instances, affecting over 63 healthcare professionals.^4^

LASV's genome is single-stranded, bipartite RNA with a large L and S gRNA, and the intergenic region divides gRNA into two coding regions. It has a spherical structure with a glycoprotein envelope and four viral proteins: glycoprotein complex (GPC), nucleoprotein (NP), matrix protein (Z), and viral polymerase (L).^5^, ^6^, ^7^ Arenaviruses have a singular polymerase protein, NP, and RNP, crucial for viral RNA replication.^8^, ^9^ The L protein of LASV plays a critical role in both the transcription and replication of the viral genetic material, while nuclear protein (NP) is equally vital for these processes. Together with L, the encapsulated RNA of the virus's genome forms the RNP. The RNA is protected from damage and detection by cellular pattern recognition receptors within the RNP. The RNP consists of a C-terminal and an N51-terminal site, which are essential for its function and structure. The N-terminal region is involved in RNA binding, whereas the C-terminal region is responsible for RNA cleavage.^9^, ^10^, ^7^ The C-terminal site of the LASV nuclear protein exhibits a close relationship with the Z protein and is essential for the assembly of ribonucleoprotein complexes (RNPs) that incorporate transmittable RNA along with L amino acids. This assembly is vital for the transcription and replication of the viral genome.^11^ NP presents a promising candidate for drug discovery from phytocompounds, as studies suggest that plant therapeutic derivatives could potentially function as LASV medications due to their strong binding affinities.^[^^4^ To find out a potential therapeutic target for LASV, NP (PDB ID: 3M5X) was selected for its critical nature in pathogenesis. The 3MX5 protein of Lassa virus (LASV) is identified as the nucleoprotein (NP) essential for virus replication and immune evasion, with DEDDh 3′-to-5′ exonuclease activity breaking down double-stranded RNA, thereby halting the host's innate immune response.^5^, ^7^ In addition to merely inhibiting protein activity, it is crucial to identify a specific phytocompound that can efficiently reduce the activity of the 3MX5 protein while concurrently preventing LASV infection. Numerous studies have utilized computational methods to identify potential antiviral agents from natural sources. Therefore, we focused on plant *Glycyrrhiza glabra* derived compounds to identify innovative therapeutics. Computational methods have shown potential in drug exploration; however, as far as we know, no studies have examined the simultaneous inhibitory effects of *Glycyrrhiza glabra* (GG) compounds on 3MX5. This study seeks to address this knowledge gap, as extracts from GG revealed a variety of bioactive compounds with antiviral properties. The entire process of developing a drug is lengthy and requires meticulous testing, highlighting the importance of utilizing computer-aided drug design to speed up the discovery phase. These methods have been utilized across a range of therapeutic domains, extending beyond the realm of antiviral drug development.^12^

**Fig. 1:**
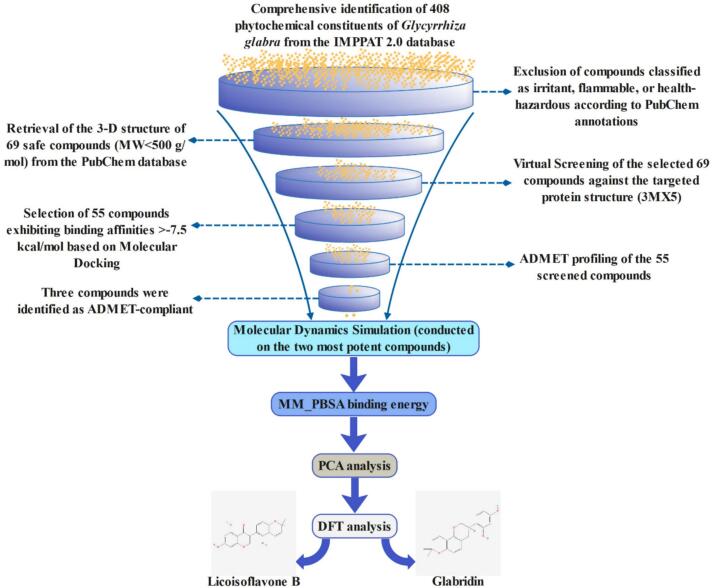
Diagrammatic representation to identify licoisoflavone B and glabridin as potential Lassa virus nucleoprotein antagonists.

Our study aims to advance the field through the application of a comprehensive computational process that incorporates molecular docking, MD simulations, binding free energy calculation, and PCA analysis. In this investigation, we initially pinpointed possible phytocompounds from *Glycyrrhiza glabra* through virtual screening utilizing 3MX5 protein based on binding energy relative to our selected control. Subsequently, by ADMET analysis, we identified compounds exhibiting superior characteristics to be a drug while excluding those with unfavorable pharmacokinetic and toxicological characteristics. Furthermore, we conducted molecular dynamics simulations and MM_PBSA analyses of the chosen protein–ligand associations to enhance our understanding of the proteins' stability and identify any structural alterations that may arise upon binding the compounds. Finally, we identified Licoisoflavone B (CID: 5481234) and Glabridin (CID: 124052) from GG as potential medicines to improve LASV treatment strategies and possibly reduce death rates, as inhibition of the 3MX5 protein demonstrates a more beneficial therapeutic effect compared to just targeting the virus. However, a notable concern is that the study primarily relies on computational methods, which, while powerful, do not account for the complex interactions and variability present in biological systems. Therefore, an *in vivo* experimental study validation is required to support these predictions. A detailed overview of the methodology and tools utilized in this study is illustrated in Fig. 1.

## 2 Methodology

### 2.1 Preparation of protein

The Lassa virus’s nucleoprotein crystal structure was retrieved from the protein data bank (RCSB) (rcsb/) with PDB ID 3MX5.^13^ The ID: 3MX5 was selected due to its higher resolution compared to other related structures, with a resolution of the protein 1.90 Å (R-Value Observed: 0.185, R-Value Free: 0.224, R-Value Work: 0.183). Subsequently, the protein underwent processing utilizing Discovery Studio 2024, which involved the removal of ligands, water particles, and heteroatoms, and also nucleic acids^14^ significantly stabilized by conducting energy depletion utilizing the GROMOS96 force field v4.10 of SWISS PDB Viewer (SPDV).^15^

### 2.2 Preparation of ligands

Plants naturally produce phytochemicals, which are chemical compounds with potential medicinal and pharmaceutical uses. Using the IMPPAT 2.0 (Indian Medicinal Plants, Phytochemistry, and Therapeutics 2.0) (imppat/) database,^16^ this research analyzes the phytochemical ingredients derived from *Glycyrrhiza glabra* and their potential negative binding effects on selected receptors. Throughout the study, we eliminated substances that raised chemical safety concerns, including corrosive, flammable, acutely toxic, irritant, and environmental hazards, according to PubChem,^17^ and incorporated all the remaining substances, which posed no safety risks and had a molecular weight of less than 500 mg/mol, into the collection. This screening procedure has led to the extraction of 69 compounds. Against the intended protein, we used a control molecule called ribavirin. It is recommended as standard treatment for LF in national and international guidelines. The 3D structure of all compounds was downloaded in SDF format from PubChem and converted into a single SDF Autodock-compatible file using OpenBabelGUI for virtual screening.^18^

### 2.3 Virtual screening and molecular docking

Virtual screening and molecular docking are common methods used in drug development to identify potential hits from a large compound library against a specific target protein. Virtual screening was carried out using PyRx 0.8 software’s^19^ Autodock_vina wizard to determine the optimal binding relationship between our target protein and the compounds.^20^, ^18^ Using the Open Babel tool in the PyRx software, all the ligands’ energy was reduced and transformed from SDF files into PDBQT files prior to docking.^19^ The 69 prepared ligands that passed ADMET analysis were docked against the target protein 3MX5. We have conducted a blind molecular docking with the values of center X = 35.0281, Y = 51.6417, and Z = -0.1402. Additionally, the dimensions (Angstrom) X = 95.2258, Y = 161.7517, and Z = 105.1770 were assigned to the grid box parameters. Using all the default parameters, exhaustiveness was set to 32 throughout the virtual screening process. The dataset was augmented before screening to include the traditional inhibitor ribavirin for control purposes. Compounds having significant binding affinities to the target protein as compared to the standard inhibitor were evaluated for further study. Discovery Studio 2024 was then used to examine the outcomes.

### 2.4 ADMET analysis

To accurately forecast how a medicine will function in the human body, the drug-like characteristics of novel drug candidates are crucial.^21^ This study utilized the SwissADME (swissadme/) web server to evaluate the physicochemical and lipophilicity characteristics of selected compounds.^22^ Following guidelines like Lipinski's,^23^ Egan's,^24^ and Veber's^25^ is necessary for determining whether chemicals are appropriate for use as drugs or not. We also assessed the physicochemical features such as molecular weight, number of H-bond acceptors, number of rotatable bonds, number of H-bond donors, molar refractivity, and the topological surface area of polar regions (TPSA). Additionally, pharmacokinetic properties, such as absorption, distribution, metabolism, and excretion (ADME) features, were predicted with the use of the online server pkCSM (pkcsm/prediction).^26^ We examined the compounds' ADME properties, such as how well they dissolved in water (log mol/L), how well they crossed the blood–brain barrier (BBB), how much they were cleared, and how well they reacted with CYP3A4 and CYP2D6. The ProTox-3.0 online server (protox3/)^27^ was utilized to forecast the toxicological evaluations regarding specific phytocompounds. Parameters like AMES toxicity, hepatotoxicity, neurotoxicity, nephrotoxicity, cardiotoxicity, carcinogenicity, mutagenicity, cytotoxicity, hERG I inhibitor (Yes/No), hERG II inhibitor (Yes/No), skin sensitisation, T. Pyriformis toxicity (log ug/L).

### 2.5 Analysis and visualization of docking results

We performed molecular docking analysis of four different compounds: ribavirin (CID: 37542), licoisoflavone B (CID: 5481234), glabrene (CID: 480774), and glabridin (CID: 124052) with their respective receptor, as they exhibited the most negative binding affinity in virtual screening. In addition, ADME, drug-likeness, and toxicity studies determined that these compounds have a high potential for success as drugs. The PyMOL Molecular Visualization System 3.0^28^ and the Discovery Studio 2024 were used to visualize and rate the optimal docked position for researching binding interactions.

### 2.6 Molecular dynamic (MD) simulation

In order to evaluate the stability of the compounds when interacting with the receptor 3MX5 protein in the physiological environment of the body, the control ribavirin (CID: 37542) and the top two pharmacological candidates, Licoisoflavone B (CID: 5481234) and Glabridin (CID: 124052), that met the ADMET criteria and exhibited higher docking scores than the control were selected for molecular dynamics simulation.^29^ The YASARA tool was used to simulate the ligand-target interaction with a minimal binding energy.^30^ The MD simulation program adjusted the protonation conditions in residues of amino acids at an approximate pH of 7.4 and a modification for the network of hydrogen bonds to increase solute stability.^30^ The complex was neutralized by adding sodium (Na^+^) and chloride (Cl^-^) ions at a concentration of 0.9 %, with an excess of either Na or Cl. With the solute represented by the AMBER14 force field,^31^ ligands by AM1BCC^32^ and GAFF2,^33^ and water by TIP3P, the simulation was authorized to continue after the steepest descent and simulated annealing minimizations to eliminate potential collisions. By using the Particle Mesh Ewald method, electrostatic forces were not subject to a threshold limit. The motion formulae were precisely implemented at 298 K ambient temperature and 1 atm atmospheric pressure, using a mean interval of 1.25 fs for bound associations and 2.5 fs for non-bonded associations.^34^ Finally, simulations were run using a Berendsen thermostat and constant air pressure for 100 ns, while snapshots were taken every 100 ps.

### 2.7 Calculation of binding free energy

The study utilized the single trajectory method to calculate binding free energy via Molecular Mechanics/Poisson-Boltzmann Surface Area (MM_PBSA), utilizing the YASARA simulator, while the AMBER14 force field and Adaptive Poisson-Boltzmann Solver were used for calculating solvation energy and electrostatics. The thermal equilibrium of NP-ribavirin, NP-licoisoflavone B, and NP-glabridin complexes was evaluated by the MM-PBSA approach. The calculation used a 10 ns molecular dynamics trajectory, derived from a constant range (normally from 90 to 100 ns) in the complexes, for the calculations. The binding free energy was calculated using the following equations:

$${\Delta G}_{\text{bind}}={\Delta G}_{\text{complex}(\text{minimized})}-\left[{\Delta G}_{\text{ligand}(\text{minimized})}+{\Delta G}_{\text{receptor}(\text{minimized})}\right]$$

$${\Delta G}_{\text{bind}}={\Delta G}_{\text{MM}}+{\Delta G}_{\text{PB}}+{\Delta G}_{\text{SA}-\;\text{T}\Delta \text{S}}$$

The formula for protein entropic participation is represented by ΔTDS, while ΔGMM represents the quantum mechanics relationship force, and ΔGPB and ΔGSA represent the polar and nonpolar solvation energies, respectively.^34^

### 2.8 PCA and DFT analysis

Principal Component Analysis (PCA) is a technique used for data wander reduction, aiming to identify similarities and differences in energy assessment of MD trajectory data. It allows for characterization of fundamental quality changes during molecular dynamics through comparison of various ligand–protein complexes.^35^ The PCA algorithm can identify unmodeled variations in the connections between the main target protein and two phytochompounds, particularly in terms of their speed, by encapsulating these residuals. This study employed PCA as a method for data reduction to identify the most important modes of motion from the extensive MD simulation trajectories. We used the last 100 ns of molecular dynamics trajectory information from three protein–ligand complexes for PCA. All calculations were conducted using Minitab 18 (minitab/) customized internal scripts, and generated plots using the Factoextra package. Finally, the DFT calculations were performed using the B3LYP/6-31G [EKHANE] (d, p) basic set of the Gaussian 09 W software. We determined the energies of the frontier molecular orbitals (HOMO, LUMO, and the energy gap), along with reactivity-related parameters such as chemical hardness, chemical softness, electronegativity, electrophilicity index, ionization potential, and electron affinity, all derived from the HOMO and LUMO values.

## 3 Results

### 3.1 Virtual screening and molecular docking

The PyRx virtual screening platform was used to reduce the number of compounds for further investigation. All the phytochemicals exhibited binding affinity scores in the range from −11.3 to −3.8 kcal/mol with the receptor. Candidates with higher negative docking scores than the control, ribavirin (−6.6 kcal/mol), with an RMSD value of 0 were selected for additional study. To assess drug-like properties, 55 compounds were chosen based on their binding affinity to protein exceeding −7.5 kcal/mol (Supplementary Table 1).

### 3.2 Evaluation of drug-likeness properties

The appropriateness of chemical substances for drug development is important in the early phases of drug discovery, as 90 % of drugs are rejected because of complications with the body's metabolism and response to medicinal products.^36^ The physicochemical and pharmacokinetic properties including, molecular weight (MW), number of rotatable bonds (NRBs), hydrogen bond acceptors (HBAs), number of hydrogen bond donors (HBDs), lipophilicity (LogP), molar refractivity (MR), topological surface area (TPSA), and bioavailability Score (BS) were assessed. It is significant to understand the relationship between these characteristics to recognize a molecule as a possible drug. After conducting the virtual evaluation, we used the SwissADME online server to analyze 55 compounds derived from *Glycyrrhiza glabra* in order to assess their potential as drugs. The findings of the assessment for the drug-like qualities obtained with the SwissADME are shown in Supplementary Table 2. To evaluate the effectiveness of oral medications, we analyzed the compounds using Lipinski's rule of five. This rule of thumb suggests that a molecule is likely to be orally available if it does not violate more than two of the specified criteria.^37^ According to Lipinski’s rule, a molecule will be considered a drug-like compound when MW ≤ 500 Daltons (Da), Log P (octanol/water) < 5 units, the number of hydrogen-bond acceptors (HBAs) ≤ 10, and the number of hydrogen-bond donors (HBDs) ≤ 5. Consequently, among 55 potential compounds, 44 satisfy all of Lipinski's criteria, 8 comply with the rule with just one permissible violation, while the rest adhere to the regulations with two violations. The Egan and Veber guidelines were also employed to evaluate these substances. Among all the drug candidates, only 7 had one permissible violation, and others did not violate the Egan rule. As for the Veber rule, all compounds met the required standards except for one rule, which was not met by 14 compounds. We have selected all the compounds that comply with Lipinski’s, Egan’s, and Veber’s rules without any violations. Each substance has a bioavailability score of 0.55, which indicates high bioavailability. After conducting drug-likeness screening, the number of molecules was reduced from 55 to 40 in order to determine whether they met ADMET requirements.

### 3.3 ADME and toxicity analysis

The importance of pharmacokinetic properties (PKs) in drug development arises from the potential for lead molecule elimination during preclinical and clinical trials due to insufficient pharmacokinetic profiles and toxicity. The properties of a successful oral medication are determined by its PK, including absorption rate from the gastrointestinal tract, transmission to the site of action, metabolism, and elimination from the body without producing any negative side effects. Estimating ADME (absorption, distribution, metabolism, and excretion) helps to predict the PK of selected compounds, which are essential for studying the effectiveness of a drug. The pkCSM server was used to evaluate a broad spectrum of ADME parameters with the aim of evaluating potential drug candidates for pharmacological implementation, in Supplementary Table 3 the results are outlined. The ADME characteristics of the drug indicated, such as their human intestinal absorption (HIA), water solubility, volume of distribution at steady state (VDss) in humans, permeability across the blood–brain barrier (BBB), substrates for CYP2D6 and CYP3A4, along with overall clearance of the drug. In order to ensure the effectiveness of a medication, it is essential to thoroughly analyze its biological activity and its ability to precisely target the specific organ at a particular dosage. The research indicates that each component exhibits a distinct spectrum of water solubility properties, which distinguishes it from others. Water solubility is a critical factor in drug discovery, as it is associated with decreased effectiveness when solubility levels are low. Compounds exhibiting values from −10 to −6 are characterized by low solubility; conversely, those with values exceeding −6 but less than −4 show moderate solubility, whereas substances falling within the range of −4 to −2 are soluble, and those with values between −2 and 0 demonstrate high solubility.^38^ Out of 40 compounds, we are focusing on 3 due to their advantageous ADMET properties. Licoisoflavone B (CID: 5481234), glabrene (CID: 480774), and glabridin (CID: 124052) as the most effective inhibitors in the dataset. These compounds have a solubility range of −3.861 to −3.544, indicating potential solubility under physiological conditions. As a result, it is observed that these compounds exhibit significant estimated absorption in the intestine of humans, exceeding 90 %. This contradicts the threshold indicating poor absorbance, which stipulates an intestinal absorption value below 30 %. This suggests that the intestinal tract may readily absorb these molecules and allow them to circulate throughout the blood.^39^ The compounds' distribution was evaluated by determining the volume of distribution in humans (VDss) and their capacity for traversing the blood–brain barrier (BBB). A VDss value of < −0.15 indicates lower distribution, while a value > 0.45 denotes higher drug distribution. These three substances exhibit a greater dispersion within the body, falling within the range of 0.505–0.619. The blood–brain barrier is a natural defense mechanism that isolates the brain from the remainder of the body, shielding it from substances carried in the bloodstream and obstructing drugs and external materials from reaching the central nervous system (CNS).^40^ According to pkCSM guidelines, these compounds are identified as potential drug candidates owing to their minimal likelihood of traversing the BBB and are regarded as possible drug candidates due to their low probability of crossing the BBB. Cytochrome P450 (P450 or CYP) enzymes, which contain heme, are predominantly located in the lipid bilayer of the hepatic endoplasmic reticulum and play a crucial role in metabolizing various compounds such as drugs, steroids, and toxins.^41^ Certain drugs act as potent inducers of enzymes, while others serve as inhibitors. Six major enzymes are thought to be responsible for 90 % of human drug oxidation (CYP types: 1A2, 2C9, 2C19, 2D6, 2E1, and 3A4/5), among them CYP3A4 and CYP2D6 are the two most important CYP isoenzymes.^42^ In the realm of metabolism, none of the substances exhibited inhibitory effects, but CID: 480,774 and CID: 124,052 were recognized as substrates for CYP3A4.

Toxicity denotes the tendency of a chemical substance to inflict harm on an organism or any of its constituent parts, such as cells and organs. Analyses of clinical trial data spanning from 2010 to 2017 revealed that inadequate management of toxicity contributed to 30 % of the failures in drug development.^43^, ^44^ Assessing toxicity is an important preliminary stage prior to subjecting a potential drug to clinical trials, and it helps enhance the selection of lead compounds. In-silico toxicity measurement using computational methods is frequently utilized because of its precision, speed, and accessibility. This approach can offer valuable insights into the toxicity of both synthesized and naturally occurring compounds. We utilized ProTox-3.0 and pkCSM to assess the toxicological profiles and potential adverse effects of the three selected compounds, aiming to bolster their viability as candidates for therapeutic applications. The findings on the possible harmful effects of all molecules under investigation are presented in Table 1. The compounds licoisoflavone B, glabrene, and glabridin, as indicated by the ProTox-3.0 server, are found to have no hepatotoxic, neurotoxic, cardiotoxic, mutagenic, carcinogenic, or cytotoxic effects. The study anticipates that none of the substances will induce AMES toxicity, hERGI, and II inhibition (except glabridin), or skin sensitization. The maximally tolerated dose refers to the highest dosage that the majority of patients can endure. Licoisoflavone B, glabrene, and glabridin were administered at doses of 0.502, −0.035, and 1.148 Log mg/kg/day, respectively, denoting moderate, low, and high levels of dosages correspondingly. The compounds were found to have oral rat acute toxicity (LD50) values of 2.397 for licoisoflavone B, 2.425 for glabrene, and 2.523 for glabridin mol/kg, indicating a satisfactory safety profile. Three compounds, licoisoflavone B, glabrene, and glabridin, met all the necessary pharmacokinetic criteria. They were discovered to have water solubility and satisfied the BBB parameter while meeting the VDss criteria for dispersion throughout the body. Additionally, none of these compounds showed inhibitory effects on any CYP isoenzymes. Furthermore, all the assessed toxicity parameters validated a significant level of safety, suggesting that these compounds are promising candidates for additional studies.

**Table 1:** Toxicity properties of the selected 3 compounds using Protox-3.0 and pkCSM.

**Server**	**Target**	**Licoisoflavone B** **(CID: 5481234)**	**Glabrene** **(CID: 480774)**	**Glabridin** **(CID: 124052)**
**Protox**	Hepatotoxicity	Inactive	Inactive	Inactive
	Neurotoxicity	Inactive	Inactive	Inactive
	Cardiotoxicity	Inactive	Inactive	Inactive
	Mutagenicity	Inactive	Inactive	Inactive
	Carcinogenicity	Inactive	Inactive	Inactive
	Cytotoxicity	Inactive	Inactive	Inactive
**pkCSM**	AMES toxicity	No	No	No
	hERG I inhibitor	No	No	No
	hERG II inhibitor	No	No	Yes
	Oral Rat Acute Toxicity (LD50)	2.397	2.425	2.523
	Skin Sensitisation	No	No	No
	Max. tolerated dose (human)	0.502	−0.035	1.148

### 3.4 Analysis of bioactivity

Bioactivity assessment usually consists of evaluating the impact of compounds on a range of biological elements, including ion channel modulation, G protein-coupled receptors (GPCR), nuclear receptor ligands, as well as inhibiting kinases, proteases, and other enzymes. This process validates the therapeutic potential of the compounds. The bioactivity score of three specified compounds was computed using the Molinspiration Cheminformatics Software. A compound is considered to have notable biological effects if its bioactivity score exceeds 0.00. Compounds with scores between −0.50 and 0.00 are expected to display moderate activity, while those with a score below −0.50 are thought to be inactive.^45^ The study demonstrates that the physiological impact of compounds can be affected by various pathways, such as interactions with GPCR ligands, nuclear receptor ligands, ion channel modulators, and enzyme inhibition. Results displayed in Table 2 indicate that the compounds exhibit moderate to high levels of activity across bioactivity parameters.

**Table 2:** Bioactivity assessments utilizing molinspiration cheminformatics software.

**Phytochemical Name**	**Compound CID**	**Parameters of Bioactivity Score**					
		**GPCR** **Ligand**	**Ion Channel Modulator**	**Kinase Inhibitor**	**Nuclear Receptor Ligand**	**Protease Inhibitor**	**Enzyme Inhibitor**
Licoisoflavone B	5,481,234	−0.05	−0.48	−0.10	0.57	−0.31	0.33
Glabrene	480,774	0.13	−0.14	−0.01	0.90	−0.02	0.35
Glabridin	124,052	0.19	−0.07	0.08	0.83	0.09	0.50

### 3.5 Protein- ligand interaction analysis

**Fig. 2:**
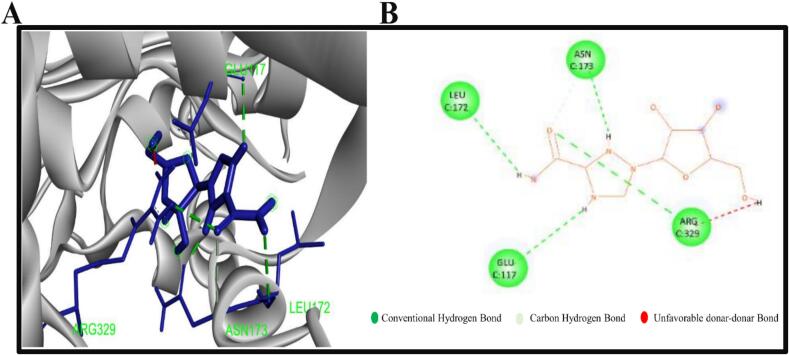
**(A)** The interaction between the control element CID37542 and 3MX5 **(B)** The 3D interaction is shown on the left of the protein ligands, while the 2D interaction is shown on the right.

**Fig. 3:**
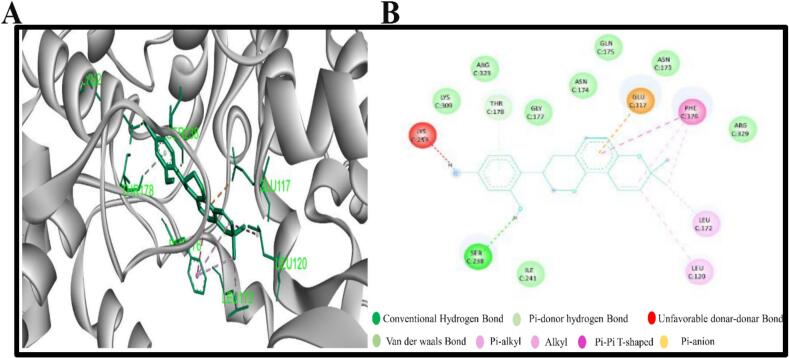
**(A)** The interaction between the chemical CID124052 and 3MX5 **(B)** The 3D interaction is shown on the left of the protein ligands, while the 2D interaction is shown on the right.

**Fig. 4:**
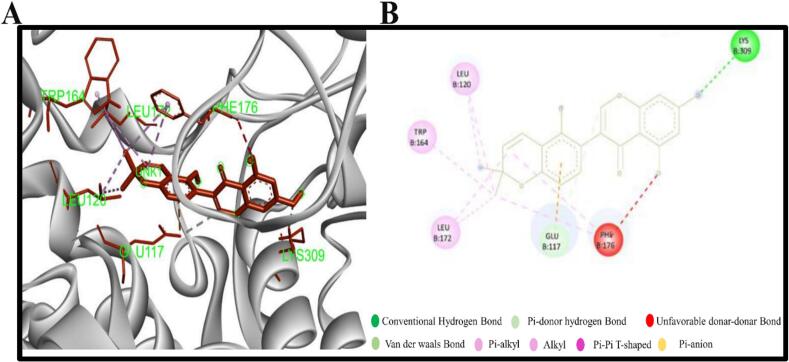
**(A)** The interaction between the chemical CID5481234 and 3MX5 **(B)** The 3D interaction is shown on the left of the protein ligands, while the 2D interaction is shown on the right.

The compounds with the more negative binding scores were selected in order to investigate potential interactions with 3MX5. The two chosen compounds were found to form a number of hydrogen and hydrophobic bonds with the target 3MX5. It has been revealed that the compound CID37542 (standard) has formed only hydrogen bonds in the GLU117, LEU172, ASN173, and ARG329 residual position, as illustrated in Fig. 2. As seen in Fig. 3 and bond types in Table 3, the compound CID124052 has additionally generated hydrophobic bonds at the LEU120 and LEU172 residual positions and hydrogen bonds at the SER238 location. The chemical CID: 5,481,234 forms several hydrogen and hydrophobic bonds with the target 3MX5. According to the bond types in Fig. 4 and Table 3, the hydrogen bonds were found to form at the position LYS309, whereas the hydrophobic bonds were formed at the positions LEU120, LEU172, and TRP164.

**Table 3:** Non bond interactions between amino acid residues of 3MX5 and selected two compounds along with ribavirin.

**ID**	**Residues**	**Distance(Å)**	**Category**	**Bond Type**
**PubChem CID: 5481234**	LYS309	2.33558	Hydrogen Bond	Conventional Hydrogen Bond
	GLU117	3.73807	Hydrogen Bond	Carbon Hydrogen Bond
	GLU117	3.53759	Electrostatic	Pi-Anion
	LEU172	4.83101	Hydrophobic	Alkyl
	LEU120	4.56469	Hydrophobic	Alkyl
	LEU172	3.71454	Hydrophobic	Alkyl
	LEU120	4.91032	Hydrophobic	Alkyl
	TRP164	4.45404	Hydrophobic	Pi-Alkyl
	PHE176	4.5496	Hydrophobic	Pi-Alkyl
	PHE176	4.44787	Hydrophobic	Pi-Alkyl
**PubChem CID: 124052**	SER238	2.23279	Hydrogen Bond	Conventional Hydrogen Bond
	GLU117	3.81656	Electrostatic	Pi-Anion
	THR178	3.3003	Hydrogen Bond	Pi-Donor Hydrogen Bond
	PHE176	4.83767	Hydrophobic	Pi-Pi T-shaped
	LEU120	5.22462	Hydrophobic	Alkyl
	LEU172	3.50292	Hydrophobic	Alkyl
	PHE176	5.15307	Hydrophobic	Pi-Alkyl
	PHE176	4.72191	Hydrophobic	Pi-Alkyl
**Ribavirin**	LEU172	2.88519	Hydrogen Bond	Conventional Hydrogen Bond
	ASN173	1.948	Hydrogen Bond	Conventional Hydrogen Bond
	GLU117	2.76209	Hydrogen Bond	Conventional Hydrogen Bond
	ARG329	2.6908	Hydrogen Bond	Conventional Hydrogen Bond
	ASN173	3.34846	Hydrogen Bond	Carbon Hydrogen Bond

### 3.6 Molecular dynamics simulation (MD)

MD simulations were used to assess the mobility of protein–ligand docking complexes throughout the time. It provides useful information on the dynamic intermolecular interactions within complexes. The interactions within the protein–ligand complex were investigated using a 100 ns MD simulation. RMSD, MSF, SASA, and H-bond data throughout the simulation period demonstrated a stable interaction between all of the chosen chemicals and the target protein.

#### 3.6.1 Root mean square deviation (RMSD)

**Fig. 5:**
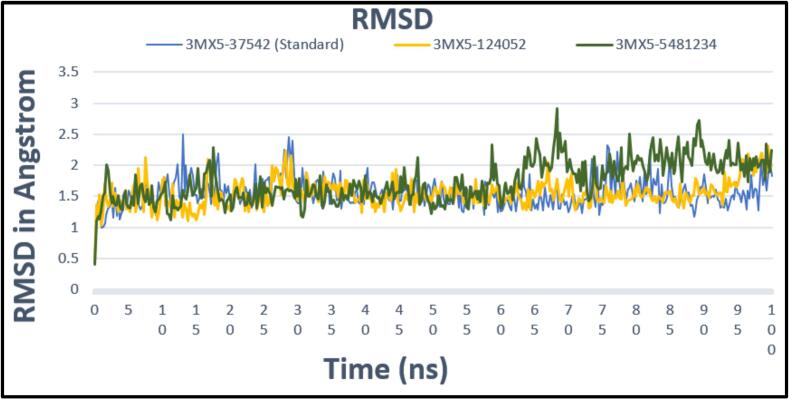
Showing the RMSD values extracted from protein ligand complexes viz, PubChem CID: 124052 (yellow), PubChem CID: 5481234 (green), where standard is marked as (light blue) concerning 100 ns simulation time.

The RMSD value of the 3MX5-CID37542 complex was determined to evaluate whether the complex remains stable or not throughout the time frame, and outcomes are demonstrated in Fig. 5. The standard complex 3MX5-CID37542 showed less deviation from its initial structure at 30 ns with an RMSD value of 2 Å, and later it remained quite stable till 100 ns, indicating that the overall backbone structure does not deviate significantly from the starting structure. The RMSD value of the first complex, 3MX5-CID124052, is comparable to the pattern of that standard complex. The second compound, the 3MX5-CID5481234 complex, showed an average of 1.5 Å RMSD value till 63 ns of the simulation period, whereas the standard showed 1.4 Å. The highest deviation was noticed for the 3MX5-CID5481234 complex from 63 to 68 ns with an RMSD value of 2.5 Å. Then, it decreased to 70 ns and remained stable in the rest of the simulation period, with an average of 2 Å. The RMSD values of nearly all were less than 3, suggesting a consistent level of complexity.

#### 3.6.2 Root mean square fluctuation (RMSF)

**Fig. 6:**
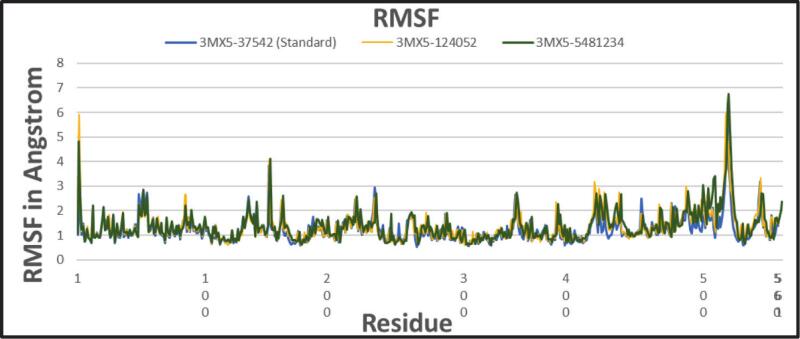
Showing the RMSF values extracted from protein ligand complexes viz, PubChem CID: 124052 (yellow), PubChem CID: 5481234 (green), where standard is marked as (light blue) concerning 100 ns simulation time.

The RMSF examines each atom's or residue's adaptability and dynamic behavior within a biomolecular system. It also influences the residual vibration in 3MX5 following ligand binding, as shown in Fig. 6. This analysis provides an overview of how ligand binding can alter protein flexibility. Our molecular dynamics (MD) simulations revealed that the highest RMSF peaks consistently mapped to residues 510–530 of the Lassa virus nucleoprotein (NP), a region located within the C-terminal exonuclease domain, directly adjacent to the canonical DEDDh active-site motif. This motif, comprising Asp389, Glu391, Asp466, Asp533, and His528, is central to the 3′–5′ exonuclease activity responsible for RNA degradation. Notably, His528 and Asp533 mark the beginning of the observed fluctuating span (∼528–533), and the adjacent loop (510–527) forms the entrance to the active-site cleft. These structural features suggest that flexibility in this loop is functionally significant, allowing ligand accommodation without destabilizing the domain. This loop exhibited a fluctuation increase of up to ∼ 6.5 Å when bound to licoisoflavone B, compared to ∼ 6 Å for ribavirin, supporting an induced-fit mechanism that optimizes ligand interaction. However, the protein with two selected phytochemicals showed an almost similar pattern in the RMSF profile compared to standard rivabirin. This suggests that the phytochemicals can sustain a consistent association without causing a major change in the structure of the proteins.

#### 3.6.3 Solvent accessible surface area (SASA)

**Fig. 7:**
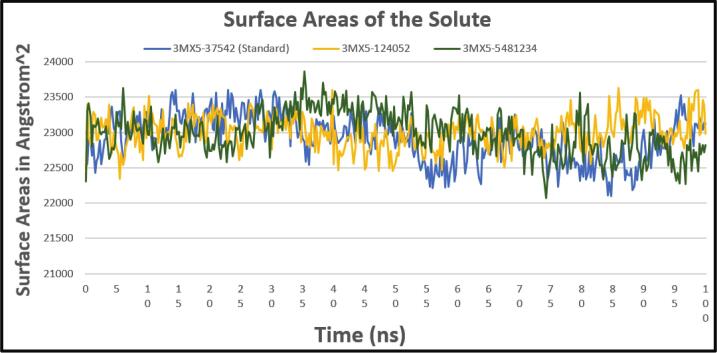
Showing the SASA values extracted from protein ligand complexes viz, PubChem CID: 124052 (yellow), PubChem CID: 5481234 (green), where standard is marked as (light blue) concerning 100 ns simulation time.

The SASA analysis determines the proportion of a molecule's external surface that is still open and available for bonding with nearby solvent molecules. This study is extremely important for understanding the protein’s integrity and folding. The average SASA value of the stanard compound is 22,900 Å2 (Fig. 7) whereas the average value of the two compounds remains constant at about 23,000 Å2. Thus, both complexs' SASA profiles showed a constant level of stability with just slight variations. It's important to note that this stability nearly matched the standard's SASA profile. This study shows that during the simulation, the solvent molecules' surface accessibility to the two complexes remained mostly constant, with just minor fluctuations, which demonstrates that the binding affinity of two selected compounds has maintained the ideal degree of protein expansion.

#### 3.6.4 Hydrogen bond analysis

**Fig. 8:**
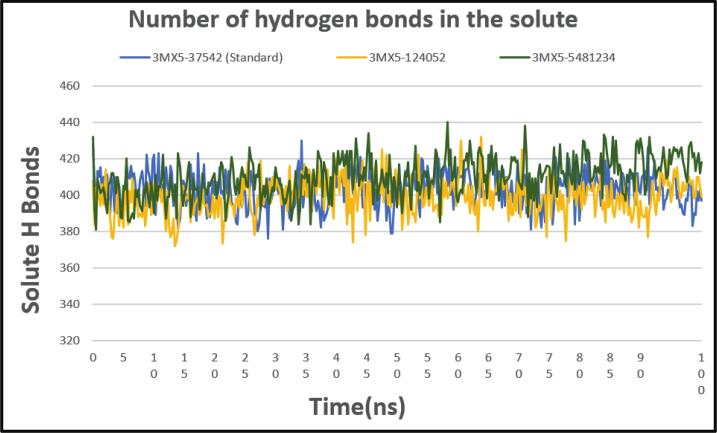
Showing the number of hydrogen bonds between solute and solvent extracted from protein ligand complexes viz, PubChem CID: 124052 (yellow), PubChem CID: 5481234 (green), where standard is marked as (light blue) concerning 100 ns simulation time.

Molecular dynamics (MD) simulation studies need hydrogen bond (H-bond) analysis because H-bonds play a crucial role in determining the structure, dynamics, and interactions of biomolecules. Based on our studies, we observed that the H-bond patterns for two complexes showed an upward and downward trend between 40 and 45 ns. The two complexes 3MX5-124052 and 3MX5-5481234 then continued to have stable H-bond profiles, which were quite similar for the duration of the 100 ns simulation. This stability was quite similar to the standard ribavirins' hydrogen bond profile. It is important to note, nevertheless, that throughout the simulation, the ordinary rivabirin showed somewhat greater hydrogen bond values than the other two complexes, 3MX5-124052 and 3MX5-5481234. The complexes subsequent stability in its H-bond profiles suggests that these complexes stabilize into configurations where their hydrogen bonding patterns remain mostly consistent over time. Additionally, complex 3MX5-124052, 3MX5-5481234, and ribavirins' comparable H-bond profiles suggest that they may share structural characteristics or interactions. All of these outcomes are presented in Fig. 8.

#### 3.6.5 Radius of gyration (Rg)

**Fig. 9:**
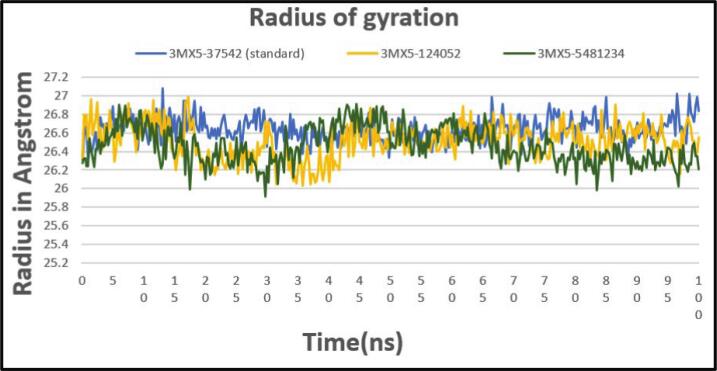
Showing the Rg values extracted from protein ligand complexes viz, PubChem CID: 124052 (yellow), PubChem CID: 5481234 (green), where standard is marked as (light blue) concerning 100 ns simulation time.

In MD simulations, RG analysis is a flexible approach that provides helpful information on the compactness and structural dynamics of biomolecules. It is essential to understanding interactions, folding processes, stability, and conformational changes, which makes molecular dynamics research a key component of drug development. A system is said to be more compact and stiff if its fluctuation rate is consistently lower throughout simulation. For the control compound, the calculated average Rg is 26.6 Å, with a maximum variation of 27.05 Å observed. Two complexes' radius of gyration (Rg) profiles initially showed a declining trend between 20 and 40 ns (ns). These profiles then remained in a consistent configuration between 40 and 70 ns. However, a steady increase in Rg values was noted following a little downward movement of about 75 ns. When compared to the standard, the first complex's Rg profiles showed a similar trend, which is very intriguing. It was discovered that, in relation to their respective time periods, both complexes displayed varying degrees of rigidity and compactness. The complexes' following stable profile from 20 to 65 ns suggests that they achieved a state that was comparatively equilibrium-like, with little alterations to their overall size and conformation. There may be common underlying processes driving the dynamics of the complexes and the standard, as indicated by the constant pattern between them (Fig. 9).

### 3.7 Calculation of binding free energy

**Fig. 10:**
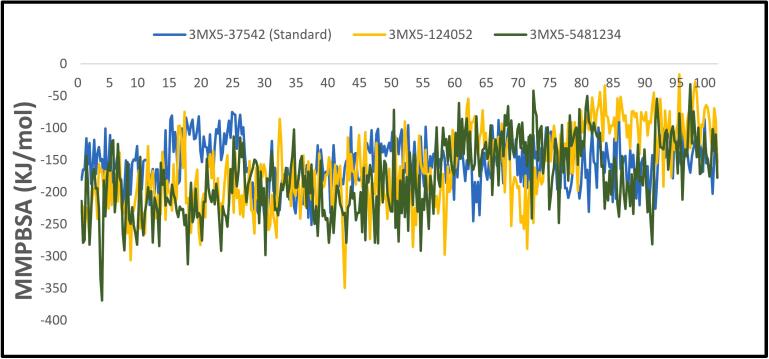
Showing the MM_PBSA values extracted from protein ligand complexes viz, PubChem CID: 124052 (yellow), PubChem CID: 5481234 (green), where standard is marked as (light blue) concerning 100 ns simulation time.

The net energy change that takes place during the formation of a protein–ligand complex in relation to the individual unbound components is quantified by the MM_PBSA binding energy calculations. Fig. 10 shows the MM_PBSA binding free energies that were calculated for three complexes during a 100 ns simulation period. Based on our research, the average MM_PBSA binding energies for the three complexes were found to be −154.303, −162.774, and −177.733 kJ/mol for 3MX5-37542 (Standard), 3MX5-124052, and 3MX5-5481234, respectively. Throughout the analysis of the 100 ns simulation period, the MM_PBSA binding energy profiles for the three complexes displayed distinct patterns of stability. Interestingly, despite this profile change, the complexes' binding energies remained mostly constant across time. In comparison to complexes 3MX5-124052 and 3MX5-5481234, these results suggest that complex 3MX5-37542.

(Standard) had a comparatively weaker binding interaction and a substantial negative MM_PBSA binding energy, revealing that complex 3MX5-5481234 had the strongest binding contact.

### 3.8 PCA and DFT analysis

**Fig. 11:**
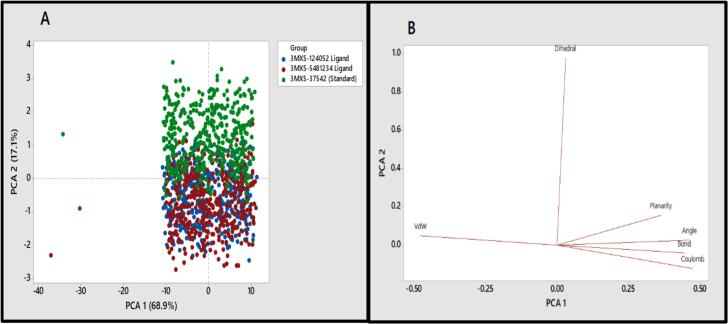
The scores plot depicted three data clusters in various colors, where each dot represented one time point. B. Loadings plot from Principal Components Analysis of the energy and structural data.

Fig. 1 displays the distribution of clusters resulting from PCA on various structural and energy variables. Each dot represents a conformer, which correlates to the structural and energetic properties that vary during the MD simulation. For the PCA model (Fig. 11A), PC1 and PC2 explain 86 % of the variation, with PC1 accounting for 68.9 % and PC2 accounting for 17.1 %. The score map reveals that the standard compound CID37542 and compound CID5481234 complexes overlap with the protein. Furthermore, except for a few time periods, the SGK1 protein complex and compounds 3, 4, and 5 overlap. However, in the positive PC1-PC2 direction, the 3MX5-37542 (Standard) complex develops its own cluster and advances away from the other classes. The bond, angle, and Van der Waals (VdW) variables all demonstrate a positive relationship with the complex, according to the PCA loading plot (Fig. 11B).

The density functional theory calculations provided key quantum chemical descriptors for ribavirin, licoisoflavone B, and glabridin (Table 4). In this analysis, glabridin exhibited the lowest band gap (3.881 eV), indicating greater chemical reactivity and enhanced charge transfer capabilities. It also showed the highest electronegativity (2.8495 eV) and electrophilicity index (2.091 eV), suggesting a strong tendency to accept electrons that is an essential property for effective ligand and receptor interaction. In contrast, licoisoflavone B had moderate values, with a band gap of 5.786 eV and an electrophilicity index of 0.1728 eV, suggesting intermediate reactivity and lower electrophilic potential compared to glabridin. However, the control ribavirin showed the highest band gap (5.997 eV) and chemical hardness (2.9985 eV), implying greater stability but lower reactivity, which could limit its interaction efficiency.

**Table 4:** Density Functional Theory (DFT)-based comparative analysis of key electronic and chemical descriptors for Ribavirin, Licoisoflavone B, and Glabridin, including ionization potential, electron affinity, electronegativity, chemical hardness, softness, electron affinity, electronegativity, chemical hardness, softness, electrophilicity index, and band gap energy.

**Compounds** **Name**	**Ionizing Potential**	**Electron Affinity**	**Electronegetivity**	**Chemical Hardness**	**Chemical Softness**	**Electrophilicity Index**	**E_Lumo_ – E_Homo_ (Band Gap)**
Ribavirin	5.482 eV	–0.515 eV	2.4835 eV	2.9985 eV	0.1668 eV	1.0287 eV	5.997 eV
Licoisoflavone B	5.476 eV	–0.310 eV	2.583 eV	2.893 eV	0.3457 eV	0.1728 eV	5.786 eV
Glabridin	4.790 eV	0.909 eV	2.8495 eV	1.9405 eV	0.2576 eV	2.091 eV	3.881 eV

## 4 Discussion

In order to combat and deal with further epidemics of the Lassa virus (LASV)-mediated illness, effective medication development is required. It would put an end to the catastrophic pandemic that was harming the people and health system of Western and Central Africa. There are currently no FDA-approved medications or vaccinations that are particular to LASV. However, ribavirin, a nonspecific antiviral that has been demonstrated to be somewhat effective against LASV infections, is the only available therapy. By today's standards, the preclinical data supporting the use of ribavirin to treat Lassa fever is contradictory and mostly insufficient to support human trials for the critical condition. Furthermore, there is no evidence of current dose guidelines for this pharmacological therapy. The infectious condition known as viral hemorrhagic fever, which is caused by LASV, poses a major risk to human health. The LASV glycoprotein complex, or GPC, is a target for anti-LASV medications and is essential to the virus's invasion process. Investigating envelope glycoprotein inhibitors may aid in the development of potent anti-LASV therapeutics. Therefore, the aim of this work was to identify and characterize a new and possibly useful bioactive antiviral medicinal molecule to inhibit the glycoprotein complex of LASV.^46^

CADD is one of the most promising ways to identify new compounds against a specific protein since it contains a number of highly advanced and nuanced features and methodologies. In this new era of contemporary drug research, CADD offers more cost-effective techniques of designing, lowers recruitment expenses, and shortens development times. It has become more well-liked and accepted among specialists in the commercial pharmaceutical industry as well as in academic circles. Molecular docking, ADMET prediction, and MD simulations are utilized in this procedure to find drug candidates with the highest biological effectiveness. Finding illness-associated proteins and researching how they interact with putative ligands are critical steps in comprehending disease processes and advancing medication discovery. Viral infections can be stopped in their tracks by finding substances that interact with and inhibit certain proteins necessary for the reproduction of viruses. By using CADD, the precise target molecule may be located by analyzing its behavior and the way the ligand binds to it. Furthermore, protein and MD simulations show the methods of protein–ligand interaction, and molecular docking pinpoints the predominant forms of binding within a ligand. Small-molecule candidates can therefore be identified as effective medications to treat specific illnesses, such as LASV infection. In order to combat and prevent the entry of LASV, fifty-five natural phytochemical compounds were screened in this study. Three of the most promising compounds were selected based on their highest binding affinity scores for additional validation based on their highest binding affinity scores. Having binding values of −10.1, −9.1, and −9.6 kcal/mol, respectively, CID: 5481234, CID: 480774, and CID: 124052 were the compounds with higher docking scores. Although CID: 5481234 is thought to have the highest target protein binding affinity among others, it also had the largest standard deviation, suggesting a larger degree of unpredictability or uncertainty in the calculation. With regard to the target protein, CID: 124052 exhibited the strongest Coulombic contact and the least amount of variability in the calculated values. The compounds that were chosen had positive traits and promising signs of their potential as drug candidates, as demonstrated by their adherence to Veber's, Egan's, and Lipinski's Rule of Five (RO5), all of which acknowledged the compounds' drug-like properties. The selected two compounds were examined, and all of them had strong ADMET characteristics. In general, the ADMET influences the drug's pharmacokinetics (PK). ADMET characteristics account for 60 % of medicinal compounds failing at the final therapeutic development phase. Early prediction of these features might significantly lower the cost of medication development. Before it can be employed as a medicine, a drug candidate must first reach its target location within the body and remain there in sufficient concentration to cause the intended biological responses. The CID: 5481234 and CID: 124052 compounds demonstrated significant levels of active intestinal absorption due to their strong water solubility index. Furthermore, CID: 5481234 and CID: 124052 were projected to have an ideal VDss score, indicating that these drug candidates can disperse in many tissues throughout the body but cannot cross the blood–brain barrier, according to pkCSM criteria. The substances CID: 5481234 and CID: 124052 can be metabolized in the liver and successfully eliminated from the body, since both were predicted not to be CYP2D6 inhibitors, while CID: 124052 and CID: 480774 were identified as CYP3A4 substrates. The most important enzymes involved in drug metabolism are CYP3A4 and CYP2D6. *In silico* toxicity study is particularly successful since it eliminates all of the disadvantages of traditional procedures, such as the need for animals, the high cost, and the time commitment associated with *in vivo* methodologies. We used the *in silico* approach to examine the toxicity profiles of the top three drugs. The statistics from the toxicity evaluation server demonstrate that none of the chemicals are carcinogenic. Furthermore, safety issues including hERG channel inhibition, neurotoxicity, cardiotoxicity, mutagenicity, cytotoxicity, hepatotoxicity, and skin sensitization were not addressed in these pharmacological candidates. While our *in silico* ADMET analysis indicates that glabridin may inhibit hERG-II channels, interestingly, a study by Huang et al. demonstrated that glabridin could prevent doxorubicin-induced cardiotoxicity in mice, suggesting potential cardioprotective effects.^47^ This finding implies that glabridin's interaction with cardiac channels may be more complex than predicted solely by *in silico* models. One of the most common reasons for medication withdrawal from clinical trials is hepatotoxicity. The study found the LD50 to be optimum as it provided information on the immediate or acute toxicity of substances. MD makes it easier to predict how two biomolecules, such as ligands and receptors, will work together to form stable complexes. With appropriate interaction models and ever-increasing computer power and availability, MD simulation has become a vital technique in the study of biophysical systems. The selected two compounds, including the control ligand, were evaluated using SASA values, RMSD, Rg, RMSF, protein–ligand contact, and ligand–protein contact. The two most prevalent metrics of structural variations are RMSD and RMSF. RMSD was measured for two compounds and a control ligand during an MD simulation, and it was discovered that the chosen compound, CID:124052, had the fewest variations when compared to the control ligand. The RMSF can estimate the locations of protein chain residues and ligand atoms. During the RMSF investigation, CID: 5481234 and CID: 124052 were the most stable molecules when compared to the control ligand. Rg represents how the atoms of a protein–ligand complex structure are organized along its axis. Following the Rg assessment, CID: 124052 is more stable than CID: 5481234, as demonstrated by our three chosen compounds. MD simulation was used to investigate the intermolecular interactions and complex structure of a protein with the provided ligands. After examination of SASA and protein–ligand interaction, it was revealed that our selected compound CID: 124052 had superior outcomes compared to the control ligand. The docking findings were validated using post-docking MM-PBSA analysis. Using principal component analysis (PCA), a statistical method, we can minimize the dimensionality of a complicated dataset while retaining the most relevant information. It is an excellent tool for protein–ligand complex research since it teaches how ligand binding affects protein dynamics and identifies the complex's primary modes of motion. According to the findings, the two chosen compounds exhibit good properties and stability while interacting with the target protein. Moreover, in DFT analysis, due to the favorable electronic descriptors, specifically the lowest band gap, highest softness, and highest electrophilicity, our selected constituents emerge as the more prominent candidate than the control compound for further investigation as a potential LASV antiviral drug.

## 5 Conclusion

Licoisoflavone B and glabridin have been recognized as promising candidates for inhibiting the LASV nucleoprotein. These two compounds demonstrated superior binding affinities compared to the control and exhibited stable interactions, as confirmed by a comprehensive computational approach that includes molecular docking, MD simulations, MM-PBSA binding free energy calculations, PCA, and DFT calculation. However, the investigation mainly utilizes computational techniques, which, although robust, fail to consider the intricate interactions and variability inherent in complex biological systems. Therefore, it is important to conduct more experiments both in vitro (binding assays, cell-based studies) and *in vivo* (in living organisms) to confirm how effective these compounds are as treatments.

## CRediT authorship contribution statement

**Sk. Faisal Ahmed:** Software, Project administration, Conceptualization. **Md. Masudur Rahman Munna:** Writing – review & editing, Writing – original draft, Validation, Supervision, Project administration. **Md. Hossain Ahmed:** Writing – review & editing, Writing – original draft, Visualization, Methodology, Formal analysis, Data curation. **Md. Mostafizur Rahman:** Writing – review & editing, Writing – original draft, Methodology, Data curation. **Minhajul Islam:** Writing – original draft, Visualization, Methodology, Formal analysis, Data curation. **Esha Mony Bristy:** Methodology, Data curation.

## Declaration of competing interest

The authors declare that they have no known competing financial interests or personal relationships that could have appeared to influence the work reported in this paper.
