# Supplementary material for: Licoisoflavone B and glabridin from Glycyrrhiza glabra as potent nucleoprotein antagonists of Lassa virus: insights from molecular docking, dynamics simulation, PCA, and DFT studies
Source: J Genet Eng Biotechnol. 2025 Aug 5;23(3):100544. doi: 10.1016/j.jgeb.2025.100544 (PMC12375212; doi:10.1016/j.jgeb.2025.100544)
Supplement: Supplementary Data 1 [file mmc1.docx]

**Table 1:** Docking score of ligands with the protein (3MX5).

| **Phytochemical Name** | **Compound CID** | **Binding Energy**  **(kcal/mol)** | **RMSD** |
| --- | --- | --- | --- |
| Glychionide A | 11597485 | -11.3 | 0 |
| Liquiritin | 503737 | -10.9 | 0 |
| 3-Hydroxyglabrol | 480854 | -10.5 | 0 |
| Shinflavanone | 197678 | -10.5 | 0 |
| Neoliquiritin | 51666248 | -10.4 | 0 |
| Glabrocoumarin | 11427657 | -10.4 | 0 |
| Glabrol | 11596309 | -9.9 | 0 |
| Hispaglabridin B | 15228661 | -9.8 | 0 |
| Neoisoliquiritin | 22524410 | -9.7 | 0 |
| licoflavone B | 11349817 | -9.6 | 0 |
| Isoliquiritin | 5318591 | -9.6 | 0 |
| Olean-12-en-29-oic acid, 3,22-dihydroxy-, I(3)-lactone, (3I(2),20I(2),22I(2))- | 101280183 | -9.5 | 0 |
| Quercetin-3-glucoside | 25203368 | -9.5 | 0 |
| 7-methoxy-3-[4-[(2S,3R,4S,5S,6R)-3,4,5-trihydroxy-6-(hydroxymethyl)oxan-2-yl]oxyphenyl]chromen-4-one | 5318619 | -9.5 | 0 |
| 7,4'-Dihydroxyflavan | 158280 | -9.5 | 0 |
| Pratol | 5320693 | -9.4 | 0 |
| Liquiritigenin | 114829 | -9.4 | 0 |
| Glycyrin | 480787 | -9.3 | 0 |
| 21alpha-Hydroxyisoglabrolide | 101280184 | -9.2 | 0 |
| liquoric acid | 101280179 | -9.2 | 0 |
| Glabroisoflavanone A | 11221431 | -9.2 | 0 |
| Licoisoflavone B | 5481234 | -9.2 | 0 |
| Pinocembrin | 68071 | -9.2 | 0 |
| Glyzarin | 44257206 | -9.1 | 0 |
| Glycyrrhisoflavone | 5317764 | -9.1 | 0 |
| Prunetin | 5281804 | -9.1 | 0 |
| Glabrene | 480774 | -9.1 | 0 |
| Kanzonol R | 131753027 | -9 | 0 |
| Glycyrrhisoflavanone | 5317762 | -9 | 0 |
| 7-Methoxy-2-methyl-3-phenyl-4H-chromen-4-one | 354368 | -9 | 0 |
| Glabrolide | 90479675 | -8.9 | 0 |
| Triterpenoids | 71597391 | -8.9 | 0 |
| Isoangustone A | 21591148 | -8.9 | 0 |
| 11-Deoxoglycyrrhetinic acid | 12305517 | -8.9 | 0 |
| Licoflavonol | 5481964 | -8.9 | 0 |
| Glabridin | 124052 | -8.9 | 0 |
| Semilicoisoflavone B | 5481948 | -8.8 | 0 |
| Glabrone | 5317652 | -8.8 | 0 |
| Hispaglabridin A | 442774 | -8.8 | 0 |
| 1-Methoxyficifolinol | 480872 | -8.7 | 0 |
| Glabranin | 124049 | -8.7 | 0 |
| Glabroisoflavanone B | 11405466 | -8.6 | 0 |
| Shinpterocarpin | 10336244 | -8.6 | 0 |
| Astragalin | 5282102 | -8.6 | 0 |
| 30-Hydroxy-11-oxo-beta-amyrin | 12310283 | -8.5 | 0 |
| Glyzaglabrin | 5317777 | -8.5 | 0 |
| 7-Hydroxy-2-methyl-3-phenyl-4H-chromen-4-one | 5380976 | -8.4 | 0 |
| Texasin | 5281812 | -8.4 | 0 |
| 7-Acetoxy-2-methylisoflavone | 268208 | -8.4 | 0 |
| Hydroxywighteone | 5378945 | -8.3 | 0 |
| Licoricone | 5319013 | -8.3 | 0 |
| Licoisoflavone A | 5281789 | -8.2 | 0 |
| (4aS,5S,8aR)-5-[2-[(1S,4aR,6S,8aR)-6-hydroxy-5,5,8a-trimethyl-2-methylidene-3,4,4a,6,7,8-hexahydro-1H-naphthalen-1-yl]ethyl]-1,1,4a,5-tetramethyl-6-methylidene-2,3,4,7,8,8a-hexahydronaphthalen-2-ol | 42608308 | -8.1 | 0 |
| Beta-Sitosterol | 222284 | -8.1 | 0 |
| Liqcoumarin | 11378967 | -7.6 | 0 |
| Ribavirin (Control) | 37542 | -6.6 | 0 |
