# Supplementary material for: Licoisoflavone B and glabridin from Glycyrrhiza glabra as potent nucleoprotein antagonists of Lassa virus: insights from molecular docking, dynamics simulation, PCA, and DFT studies
Source: J Genet Eng Biotechnol. 2025 Aug 5;23(3):100544. doi: 10.1016/j.jgeb.2025.100544 (PMC12375212; doi:10.1016/j.jgeb.2025.100544)
Supplement: Supplementary Data 2 [file mmc2.docx]

**Table 2:** Evaluation of the physicochemical properties, lipophilicity, and drug-likeness parameters of the chosen compounds using the SwissADME online server.

| **Phytochemical Name** | **Compound CID** | **Drug Likeness Parameters** | | | | | | | **Violation** | | |
| --- | --- | --- | --- | --- | --- | --- | --- | --- | --- | --- | --- |
|  |  | **MW (g/mol)** | **nRB** | **nHBA** | **nHBD** | **MR** | **TPSA (Å²)** | **iLOGP** | **L** | **V** | **E** |
| Glychionide A | 11597485 | 446.36 | 4 | 11 | 6 | 106.72 | 187.12 | 1.63 | 2 | 1 | 1 |
| Liquiritin | 503737 | 418.39 | 4 | 9 | 5 | 101.67 | 145.91 | 2.22 | 0 | 1 | 1 |
| 3-Hydroxyglabrol | 480854 | 408.49 | 5 | 5 | 3 | 118.15 | 86.99 | 3.25 | 0 | 0 | 0 |
| Shinflavanone | 197678 | 390.47 | 3 | 4 | 1 | 115.37 | 55.76 | 4.17 | 0 | 0 | 0 |
| Neoliquiritin | 51666248 | 418.39 | 4 | 9 | 5 | 101.67 | 145.91 | 1.95 | 0 | 1 | 1 |
| Glabrocoumarin | 11427657 | 336.34 | 1 | 5 | 2 | 96.09 | 79.90 | 2.57 | 0 | 0 | 0 |
| Glabrol | 11596309 | 392.49 | 5 | 4 | 2 | 116.99 | 66.76 | 4.04 | 0 | 0 | 0 |
| Hispaglabridin B | 15228661 | 390.47 | 1 | 4 | 1 | 115.35 | 47.92 | 4.13 | 0 | 0 | 0 |
| Neoisoliquiritin | 22524410 | 418.39 | 6 | 9 | 6 | 104.44 | 156.91 | 2.57 | 1 | 1 | 1 |
| Licoflavone B | 11349817 | 390.47 | 5 | 4 | 2 | 119.41 | 70.67 | 4.04 | 0 | 0 | 1 |
| Isoliquiritin | 5318591 | 418.39 | 6 | 9 | 6 | 104.44 | 156.91 | 2.64 | 1 | 1 | 1 |
| Deoxoglabrolide | 101280183 | 454.68 | 0 | 3 | 1 | 134.05 | 46.53 | 4.09 | 1 | 0 | 1 |
| quercetin-3-glucoside | 25203368 | 463.37 | 4 | 12 | 7 | 108.27 | 213.34 | 1.22 | 2 | 1 | 1 |
| 7,4'-Dihydroxyflavan | 158280 | 242.27 | 1 | 3 | 2 | 69.13 | 49.69 | 2.07 | 0 | 0 | 0 |
| Pratol | 5320693 | 268.26 | 2 | 4 | 1 | 76.43 | 59.67 | 2.45 | 0 | 0 | 0 |
| Liquiritigenin | 114829 | 256.25 | 1 | 4 | 2 | 69.55 | 66.76 | 1.73 | 0 | 0 | 0 |
| Glycyrin | 480787 | 382.41 | 5 | 6 | 2 | 108.67 | 89.13 | 3.31 | 0 | 0 | 0 |
| 21alpha-Hydroxyisoglabrolide | 101280184 | 484.67 | 0 | 5 | 2 | 135.45 | 83.83 | 3.43 | 0 | 0 | 0 |
| liquoric acid | 101280179 | 484.67 | 1 | 5 | 2 | 135.82 | 83.83 | 3.33 | 0 | 0 | 0 |
| Glabroisoflavanone A | 11221431 | 338.35 | 1 | 5 | 2 | 93.67 | 75.99 | 2.62 | 0 | 0 | 0 |
| Licoisoflavone B | 5481234 | 352.34 | 1 | 6 | 3 | 98.11 | 100.13 | 2.89 | 0 | 0 | 0 |
| Pinocembrin | 68071 | 256.25 | 1 | 4 | 2 | 69.55 | 66.76 | 2.11 | 0 | 0 | 0 |
| Glyzarin | 44257206 | 294.30 | 2 | 4 | 1 | 85.10 | 67.51 | 2.35 | 0 | 0 | 0 |
| Glycyrrhisoflavone | 5317764 | 354.35 | 3 | 6 | 4 | 99.73 | 111.13 | 2.56 | 0 | 0 | 0 |
| Prunetin | 5281804 | 284.26 | 2 | 5 | 7 | 78.46 | 79.90 | 2.50 | 0 | 0 | 0 |
| Glabrene | 480774 | 322.35 | 1 | 4 | 2 | 94.36 | 58.92 | 2.79 | 0 | 0 | 0 |
| Kanzonol R | 131753027 | 370.44 | 5 | 5 | 2 | 105.83 | 68.15 | 3.74 | 0 | 0 | 0 |
| Glycyrrhisoflavanone | 5317762 | 368.38 | 2 | 6 | 2 | 100.16 | 85.22 | 2.80 | 0 | 0 | 0 |
| 7-Methoxy-2-methyl-3-phenyl-4H-chromen-4-one | 354368 | 266.29 | 2 | 3 | 0 | 79.38 | 39.44 | 3.01 | 0 | 0 | 0 |
| Glabrolide | 90479675 | 468.67 | 0 | 4 | 1 | 134.25 | 63.60 | 3.79 | 1 | 0 | 0 |
| triterpenoids | 71597391 | 472.66 | 1 | 5 | 4 | 133.99 | 97.99 | 3.15 | 0 | 0 | 0 |
| Isoangustone A | 21591148 | 422.47 | 5 | 6 | 4 | 123.45 | 111.13 | 3.85 | 0 | 0 | 0 |
| 11-Deoxoglycyrrhetinic acid | 12305517 | 456.70 | 1 | 3 | 2 | 136.65 | 57.53 | 3.78 | 1 | 0 | 1 |
| Licoflavonol | 5481964 | 354.35 | 3 | 6 | 4 | 99.73 | 111.13 | 2.93 | 0 | 0 | 0 |
| Glabridin | 124052 | 324.37 | 1 | 4 | 2 | 93.25 | 58.92 | 2.97 | 0 | 0 | 0 |
| Semilicoisoflavone B | 5481948 | 352.34 | 1 | 6 | 3 | 98.11 | 100.13 | 3.05 | 0 | 0 | 0 |
| Glabrone | 5317652 | 336.34 | 11 | 5 | 2 | 96.09 | 79.90 | 2.78 | 0 | 0 | 0 |
| Hispaglabridin A | 442774 | 392.49 | 3 | 4 | 2 | 116.97 | 58.92 | 4.21 | 0 | 0 | 0 |
| 1-Methoxyficifolinol | 480872 | 422.51 | 5 | 5 | 2 | 122.63 | 68.15 | 4.20 | 0 | 0 | 0 |
| Glabranin | 124049 | 324.37 | 3 | 4 | 2 | 93.27 | 66.76 | 2.99 | 0 | 0 | 0 |
| Glabroisoflavanone B | 11405466 | 352.38 | 2 | 5 | 1 | 98.14 | 64.99 | 2.89 | 0 | 0 | 0 |
| Shinpterocarpin | 10336244 | 322.35 | 0 | 4 | 1 | 90.79 | 47.92 | 3.09 | 0 | 0 | 0 |
| Astragalin | 5282102 | 448.38 | 4 | 11 | 7 | 108.13 | 190.28 | 0.53 | 2 | 1 | 1 |
| Glycyrrhetol | 12310283 | 456.70 | 1 | 3 | 2 | 136.24 | 57.53 | 4.11 | 1 | 0 | 1 |
| Glyzaglabrin | 5317777 | 298.25 | 1 | 6 | 2 | 78.03 | 89.13 | 2.23 | 0 | 0 | 0 |
| 7-Hydroxy-2-methyl-3-phenyl-4H-chromen-4-one | 5380976 | 252.26 | 1 | 3 | 1 | 74.91 | 50.44 | 2.36 | 0 | 0 | 0 |
| Texasin | 5281812 | 284.26 | 2 | 5 | 2 | 78.46 | 79.90 | 2.39 | 0 | 0 | 0 |
| 7-Acetoxy-2-methylisoflavone | 268208 | 294.30 | 3 | 4 | 0 | 84.38 | 56.51 | 3.01 | 0 | 0 | 0 |
| Hydroxywighteone | 5378945 | 354.35 | 4 | 6 | 4 | 98.87 | 111.13 | 2.44 | 0 | 0 | 0 |
| Licoricone | 5319013 | 382.41 | 5 | 6 | 2 | 108.67 | 89.13 | 3.24 | 0 | 0 | 0 |
| Licoisoflavone A | 5281789 | 354.35 | 3 | 6 | 4 | 99.73 | 111.13 | 2.85 | 0 | 0 | 0 |
| 8(26),14(27)-onoceradiene-3beta,21alpha-diol | 42608308 | 456.74 | 3 | 2 | 2 | 142.75 | 40.46 | 4.67 | 1 | 0 | 1 |
| Beta-sitosterol | 222284 | 414.71 | 6 | 1 | 1 | 133.23 | 20.23 | 4.79 | 1 | 0 | 1 |
| Liqcoumarin | 11378967 | 218.21 | 1 | 4 | 1 | 59.67 | 67.51 | 2.05 | 0 | 0 | 0 |
| 7-methoxy-3-[4-[(2S,3R,4S,5S,6R)-3,4,5-trihydroxy-6-(hydroxymethyl) oxan-2-yl]oxyphenyl]chromen-4-one | 5318619 | 430.40 | 5 | 9 | 4 | 108.56 | 138.82 | 2.09 | 0 | 0 | 1 |
| **MW** (Molecular Weight) **< 500**, **nRB** (Number of Rotatable Bond) **< 10**, **nHBA** (Number of Hydrogen Bond Acceptor) **< 10**, **nHBD** (Number of Hydrogen Bond Donor) **< 5**, **MR** (Molar Refractivity) **≥40 - ≤130**, **TPSA** (Topological Polar Surface Area) **≤ 140**, **iLOGP** (Lipophilicity) **< 5**, **L**(Lipinski’s) **≤ 1**, **V** (Veber) ≤ **1**, **E** (Egan) **≤ 1** | | | | | | | | | | | |
