# Supplementary material for: Licoisoflavone B and glabridin from Glycyrrhiza glabra as potent nucleoprotein antagonists of Lassa virus: insights from molecular docking, dynamics simulation, PCA, and DFT studies
Source: J Genet Eng Biotechnol. 2025 Aug 5;23(3):100544. doi: 10.1016/j.jgeb.2025.100544 (PMC12375212; doi:10.1016/j.jgeb.2025.100544)
Supplement: Supplementary Data 3 [file mmc3.docx]

**Table 3:** ADME properties of selected ligands from the library of *Glycyrrhiza glabra* plant natural products.

| **Phytochemical Name** | **Compound CID** | **Absorption** | | **Distribution** | | **Metabolism** | | |
| --- | --- | --- | --- | --- | --- | --- | --- | --- |
|  |  | **Water Solubility** | **Intestinal Absorption**  **(Human)** | **Volume of Distribution**  **(Human)** | **Blood-Brain Barrier**  **Permeability** | **CYP** | | |
|  |  |  |  |  |  | **2D6** | **3A4** | **2D6** |
|  |  |  |  |  |  | **Substrate** | | **Inhibitor** |
|  |  | **Numeric (log mo1/L)** | **Numeric (% Absorbed)** | **(Log L/Kg)** | **Numeric (log BB)** | **Categorical (Yes/No)** | | |
| 3-Hydroxyglabrol | 480854 | -4.582 | 90.973 | -0.194 | -0.65 | No | Yes | No |
| Shinflavanone | 197678 | -6.226 | 93.109 | 0.411 | 0.05 | No | Yes | No |
| Glabrocoumarin | 11427657 | -4.005 | 93.947 | 0.328 | -0.182 | No | Yes | No |
| Glabrol | 11596309 | -5.521 | 90.398 | -0.055 | -0.161 | No | Yes | No |
| Hispaglabridin B | 15228661 | -5.346 | 94.077 | 0.589 | -0.027 | No | Yes | No |
| 7,4'-Dihydroxyflavan | 158280 | -3.451 | 92.538 | 0.44 | 0.12 | No | Yes | No |
| Pratol | 5320693 | -3.402 | 95.803 | -0.077 | 0.303 | No | Yes | No |
| Liquiritigenin | 114829 | -3.304 | 94.333 | 0.218 | 0.375 | No | No | No |
| Glycyrin | 480787 | -4.024 | 93.555 | 0.111 | -0.54 | No | Yes | No |
| 21alpha-Hydroxyisoglabrolide | 101280184 | -5.601 | 95.332 | 0.092 | -0.095 | No | Yes | No |
| liquoric acid | 101280179 | -4.144 | 95.541 | -0.532 | -0.059 | No | Yes | No |
| Glabroisoflavanone A | 11221431 | -3.386 | 95.291 | 0.442 | -0.178 | No | Yes | No |
| Licoisoflavone B | 5481234 | -3.544 | 90.671 | 0.505 | -0.89 | No | No | No |
| Pinocembrin | 68071 | -3.538 | 92.417 | -0.386 | 0.42 | No | No | No |
| Glyzarin | 44257206 | -3.736 | 96.249 | -0.232 | 0.029 | No | Yes | No |
| Glycyrrhisoflavone | 5317764 | -3.235 | 88.375 | 0.292 | -0.95 | No | Yes | No |
| Prunetin | 5281804 | -3.499 | 95.535 | -0.066 | -0.32 | No | Yes | No |
| Glabrene | 480774 | -3.861 | 92.229 | 0.619 | 0.107 | No | Yes | No |
| Kanzonol R | 131753027 | -4.82 | 91.442 | 0.124 | -0.042 | No | Yes | No |
| Glycyrrhisoflavanone | 5317762 | -3.65 | 95.802 | 0.354 | -0.374 | No | Yes | No |
| 7-Methoxy-2-methyl-3-phenyl-4H-chromen-4-one | 354368 | -4.291 | 98.574 | -0.017 | 0.382 | No | Yes | No |
| Triterpenoids | 71597391 | -3.061 | 63.438 | -1.357 | -0.801 | No | Yes | No |
| Isoangustone A | 21591148 | -3.576 | 89.78 | -0.464 | -1.025 | No | Yes | No |
| Licoflavonol | 5481964 | -3.308 | 85.395 | 0.477 | -1.125 | No | Yes | No |
| Glabridin | 124052 | -3.646 | 94.164 | 0.613 | 0.087 | No | Yes | No |
| Semilicoisoflavone B | 5481948 | -3.485 | 98.022 | 0.486 | -0.937 | No | Yes | No |
| Glabrone | 5317652 | -3.635 | 96.194 | 0.394 | 0.031 | No | Yes | No |
| Hispaglabridin A | 442774 | -4.649 | 92.326 | 0.485 | -0.364 | No | Yes | No |
| 1-Methoxyficifolinol | 480872 | -4.361 | 91.806 | 0.026 | 0.185 | No | Yes | No |
| Glabranin | 124049 | -3.916 | 92.071 | -0.021 | -0.347 | No | Yes | No |
| Glabroisoflavanone B | 11405466 | -4.85 | 92.262 | 0.018 | -0.29 | No | Yes | No |
| Shinpterocarpin | 10336244 | -3.694 | 96.183 | 0.385 | 0.239 | No | Yes | No |
| Glyzaglabrin | 5317777 | -3.165 | 96.225 | -0.177 | -0.508 | No | Yes | No |
| 7-Hydroxy-2-methyl-3-phenyl-4H-chromen-4-one | 5380976 | -3.655 | 95.529 | -0.331 | 0.313 | No | Yes | No |
| Texasin | 5281812 | 3.746 | 93.17 | -0.346 | -0.262 | No | Yes | No |
| 7-Acetoxy-2-methylisoflavone | 268208 | -4.411 | 99.189 | -0.116 | -0.048 | No | Yes | No |
| Hydroxywighteone | 5378945 | -3.563 | 80.699 | 0.238 | -1.055 | No | Yes | No |
| Licoricone | 5319013 | -4.058 | 96.082 | 0.09 | -0.55 | No | Yes | No |
| Licoisoflavone A | 5281789 | -3.31 | 80.93 | 0.434 | -0.949 | No | No | No |
| Liqcoumarin | 11378967 | -2.464 | 94.495 | -0.135 | -0.124 | No | No | No |
